# Do Chest Pain Characteristics in Patients with Acute Myocardial Infarction Differ between Those with and without Obstructive Coronary Artery Disease?

**DOI:** 10.3390/jcm12144595

**Published:** 2023-07-10

**Authors:** Sivabaskari Pasupathy, Sarena La, Rosanna Tavella, Christopher Zeitz, Matthew Worthley, Ajay Sinhal, Margaret Arstall, John F. Beltrame

**Affiliations:** 1 School of Medicine, Faculty of Health Sciences, The University of Adelaide, Adelaide, SA 5000, Australia; sivabaskari.pasupathy@adelaide.edu.au (S.P.);; 2 Central Adelaide Local Health Network, Adelaide, SA 5000, Australia; 3 Basil Hetzel Institute for Translational Health Research, Adelaide, SA 5011, Australia; 4 Southern Adelaide Local Health Network, Adelaide, SA 5042, Australia; 5 School of Medicine, Faculty of Health Sciences, Flinders University, Adelaide, SA 5042, Australia; 6 Northern Adelaide Local Health Network, Adelaide, SA 5112, Australia

**Keywords:** myocardial infarction, coronary angiography, MINOCA, chest pain, myocardial infarction with non-obstructive coronary arteries

## Abstract

The universal definition of acute myocardial infarction (MI) requires both evidence of myocardial injury and myocardial ischaemia. In MINOCA (MI with non-obstructive coronary arteries), patients must fulfil this MI criteria, but is their chest pain similar to those who have MI with obstructive CAD (MICAD)? This study compares prospectively collected chest pain features between patients with MINOCA and MICAD. Utilising the Coronary Angiogram Database of South Australia (CADOSA), consecutive MI patients were categorized as MINOCA or MICAD based on angiographic findings. Chest pain data were collected via direct patient interviews by trained staff members. Of 6811 consecutive patients fulfilling a clinical MI diagnosis, 411 (6.0%) were MINOCA, and 5948 MICAD. The MINOCA patients were younger, more often female and had less cardiovascular risk factors than those with MICAD. There were no significant differences in chest pain characteristics between the MINOCA and MICAD cohorts in relation to pain location, quality, associated symptoms, or duration. In conclusion, MINOCA patients have chest pain characteristics that are indistinguishable from MICAD patients, suggesting that their pain is ischaemic in nature. Thus, in the presence of positive myocardial injury markers, ischaemic chest pain fulfils the universal criteria for MI, despite the absence of obstructive coronary artery disease.

## 1. Introduction

The universal definition of acute myocardial infarction (MI) is based upon the demonstration of acute myocardial injury (e.g., troponin rise/fall) in the context of myocardial ischaemia (i.e., symptoms and/or diagnostic ECG/imaging changes) [1]. In contemporary clinical practice, these patients typically undergo invasive coronary angiography to determine the extent of coronary artery disease (CAD) and whether revascularization is warranted. When angiography demonstrates obstructive CAD (i.e., MICAD, myocardial infarction with obstructive CAD), the attending clinicians do not question the diagnosis of acute MI due to obstructive CAD, and manage the patient appropriately. However, if the same patient does not demonstrate obstructive CAD on coronary angiography, then a myriad of diagnoses are considered, from ‘false positive myocardial infarct’ (suggesting the troponin changes are an aberration) to non-ischaemic causes (such as myocarditis or takotsubo syndrome), and in some, myocardial infarction with non-obstructive coronary arteries (MINOCA). Hence, the coronary angiography is a key decision-making intervention, but it may also bias the clinician into dismissing the patient as not having a significant pathological condition, despite the acute coronary syndrome presentation.

MINOCA accounts for 5–10% of acute myocardial infarct presentations [2]. Its diagnostic criteria include (1) universal definition of MI, (2) the absence of obstructive coronary artery disease—i.e., no lesion ≥50%, and (3) no clinically overt specific cause for the acute presentation [3,4]. Accordingly, patients with classical presentations of myocarditis or takotsubo syndrome should not be diagnosed as MINOCA [3,5]. Cardiac magnetic resonance (CMR) imaging can distinguish more subtle presentations of these MINOCA mimickers; however, the limited availability of this resource-intensive modality is a constraint. For patients with confirmed MINOCA, further investigation is essential to elucidate the underlying coronary mechanisms responsible for their MI, such as coronary spasm or coronary microvascular dysfunction. This knowledge is crucial in initiating the appropriate therapy [4,6].

A key challenge in the diagnosis of MINOCA is interpreting the universal definition of MI, especially considering the central role of the troponin biomarker and its ‘organ-specific’ (rather than ‘disease-specific’) properties in diagnosing MI [4]. Thus, the fourth universal definition of MI [1] requires the presence of both myocardial injury and myocardial ischaemia, where myocardial injury is defined as a rise and/or fall in serial troponin measurements, with at least one value exceeding the 99th percentile of the upper reference range; myocardial ischaemia is confirmed on the basis of ischaemic symptoms, ischaemic ECG changes (such as ST/T wave changes or new Q waves), the presence of a coronary thrombus, a new MI perfusion defects, or a new regional wall motion abnormality. If a patient fulfils these criteria, then a MI should be diagnosed, irrespective of the presence or absence of CAD on coronary angiography. When there is objective evidence of myocardial ischaemia on ECG/imaging studies associated with a rising troponin, diagnosing MI is straightforward. However, if the only ischaemic marker associated with the rising troponin levels is ‘ischaemic chest pain’, then clinicians will often question whether the pain is ‘ischaemic’, and thus the diagnostic criteria may not be fulfilled. Thus the question arises as to whether chest pain in patients with MINOCA is similar to that in those with MICAD?

The primary objective of this study is to compare chest pain features in consecutive patients fulfilling the fourth set of universal criteria for MI with (MICAD) and without (MINOCA) obstructive coronary artery disease. The secondary objective is to determine if chest pain features differ between MICAD and MINOCA in those who also have distinctive ischaemic ECG changes (i.e., STEMI—ST Elevation MI), as well as those with less specific ECG changes (i.e., NSTEMI—Non ST Elevation MI).

## 2. Materials and Methods

The study population was selected from the Coronary Angiogram Database of South Australia (CADOSA) registry.

### 2.1. Data Source

CADOSA is a statewide prospective registry of consecutive patients undergoing diagnostic coronary angiography in South Australian public hospitals (population 1.6 million). The registry follows data elements and definitions (see Supplementary Table S1 for cardiovascular risk factor definitions) that align with the American College of Cardiology’s National Cardiovascular Data (NCDR)^®^ CathPCI^®^ Registry [7,8]. The NCDR CathPCI Registry is a renowned national quality improvement data registry established by the American College of Cardiology and the Society for Cardiovascular Angiography and Interventions.

A central database management centre oversees the operation of the CADOSA registry and conducts regular data audits. To validate the acute MI diagnosis, 100% of patients identified with troponin-positive non-obstructive coronary arteries (TNOCA) [9], and 10% of patients identified as MICAD were independently audited to ensure consistency with the universal definition of acute MI. The institutional human research ethics committee of each participating hospital approved the study.

The CADOSA registry data collection was undertaken by trained professional data abstractors based at each hospital site, who (where possible) obtained the required data elements via direct patient interviews and/or case note extraction. In addition to standard cardiovascular risk factors, comorbidities, coronary angiographic findings, and discharge medications, detailed chest pain characteristics were obtained. This includes pain location (substernal, left side of the chest, right side of the chest, arm, jaw, etc.), quality (tightness, heaviness, burning, squeezing, etc.), precipitating factors (physical activity, emotional stress, etc.), relieving factors (rest, nitrates, etc.), associated symptoms (sweating, nausea, etc.) and duration (≤15 s, > 15 s ≤ 15 min and > 15 min ≤ 30 min, etc.). Details of these characteristics are illustrated in Supplementary Figure S1.

### 2.2. Patient Criteria

The study cohort included consecutive acute MI patients hospitalized between January 2012 and December 2015 undergoing coronary angiography.

The inclusion criteria for acute MI were based upon the fourth set of universal criteria for MI [1], with documentation of (1) acute myocardial injury, i.e., a positive cardiac biomarker (troponin T) exceeding the upper limit of normal according to institutional laboratory parameters, with serial measurements confirming a rise or fall; and (2) the presence of an ischaemic marker such as (i) chest pain, (ii) ischaemic ECG changes (ST/T wave changes or new Q waves), (iii) MI perfusion imaging markers, (iv) new regional wall motion on ventricular imaging, or (v) coronary artery thrombus. ECG features were further classified as STEMI with new or presumed ST segment elevation or new left bundle branch block not resolved within 20 min, and NSTEMI with an absence of diagnostic ST elevation. Based upon angiographic findings, the MI patients were categorized into MICAD (presence of ≥50% stenosis) or MINOCA (no coronary lesions ≥50% stenosis).

The exclusion criteria included (i) out-of-hospital cardiac arrest; (ii) prior coronary artery bypass grafting or a percutaneous coronary intervention (PCI) within the preceding 6 months in MINOCA cohort; and (iii) a discharge diagnosis of takotsubo cardiomyopathy, myocarditis, pulmonary embolism, or other non-ischaemic causes as per the treating cardiologist/additional investigation, such as cardiac magnetic resonance imaging or echocardiogram. To ensure non-ischaemic causes were excluded in the MINOCA patient cohort, a comprehensive medical record review of all patients with suspected MI and non-obstructive coronaries was undertaken by SP and RT, with equivocal cases independently adjudicated by JB.

### 2.3. Data Analysis

The study parameters were described using frequencies and percentages for categorical variables, and median and interquartile ranges (IQR) for continuous data. Comparison between groups for categorical outcomes (clinical and chest pain characteristics, in-hospital outcomes, and discharge management) was undertaken using logistic or linear regression with MINOCA as the binary independent variable. Analyses were age- and gender-adjusted where appropriate, and the final odds ratios are reported with 95% confidence intervals. Statistical significance was established at an alpha level of 0.05. All statistical analyses were performed using Stata statistical software suite (STATA/MP version 17.0 for Mac (Apple Silicon) Revision 8 March 2023; StataCorp., LP, College Station, TX, USA).

## 3. Results

**Figure 1:**
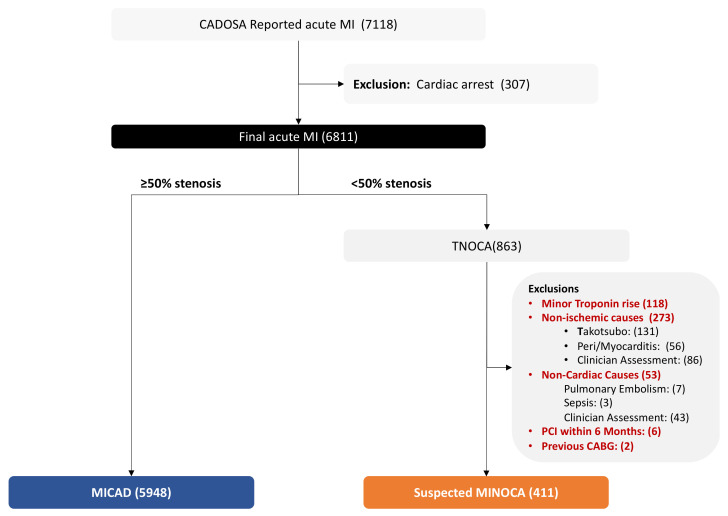
Study selection process. Minor troponin rise was audited for TNOCA patients and is defined as the following: (i) only one troponin value available and below <100 ng/L; (ii) no rise and/or fall in cardiac troponin values > 100 ng/L. CABG, coronary artery bypass grafting; MI, myocardial infarction; MICAD, myocardial infarction with coronary artery disease; PCI; percutaneous coronary intervention; MINOCA, myocardial infarction with non-obstructive coronary artery disease; TNOCA, troponin-positive with non-obstructive coronary arteries.

Within the CADOSA Registry, from January 2012 to December 2015, there were 7118 consecutive acute MI presentations who underwent coronary angiography. Of these, 307 were excluded because of out-of-hospital cardiac arrest, and 452 cases were excluded due to clinical features suggestive of causes other than MINOCA. (Figure 1). A total of 5948 patients were identified as MICAD, and 411 patients as suspected MINOCA.

### 3.1. Clinical Risk Factors and Comorbidities

MINOCA patients were younger, more likely to be female, and had fewer cardiovascular risk factors compared to MICAD (Table 1). There was no difference between groups in the prevalence of co-morbidities including heart failure, valvular heart disease, stroke, dialysis, depression, or gastroesophageal reflux disease. However, MINOCA patients were less likely to have suffered a prior MI or experienced any chest pain in the 2 weeks before their index MI, compared to those with MICAD (Table 1). The MINOCA patients were also less likely to present with STEMI and had lower peak troponin values (Table 1). In addition, there was a longer delay from pain onset to medical facility arrival for patients with MINOCA compared with MICAD (i.e., median times: 324 min [IQR 1111] vs. 280 min [IQR 872], respectively, *p* < 0.05). Finally, at discharge, MINOCA patients were less likely to receive secondary cardioprotective therapies or referral to a cardiac rehabilitation program (Table 1).

### 3.2. Chest Pain Characteristics

**Figure 2:**
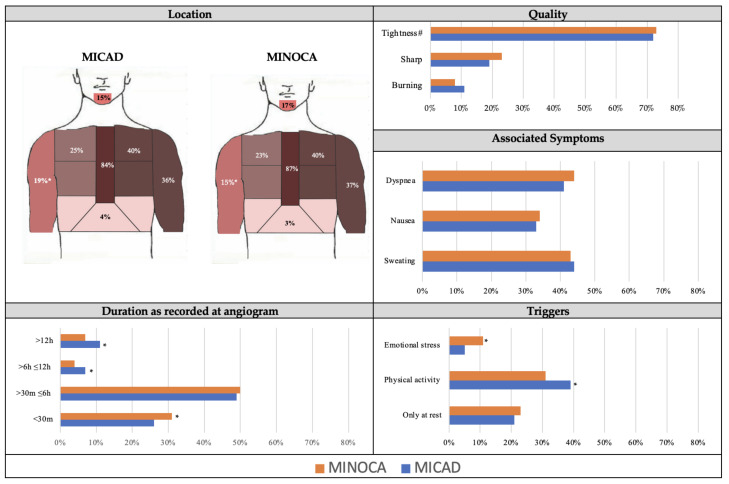
Chest pain characteristics of MICAD and MINOCA patients. MICAD, myocardial infarction with coronary artery disease; MINOCA, myocardial infarction with non-obstructive coronary arteries; h, hours; m, minutes. # Tightness is a pooled analysis including the chest pain characteristics of tightness, heaviness and squeezing. * *p*-value <0.05.

As detailed in Figure 2, there were no differences between MICAD and MINOCA patients in chest pain location, quality, associated symptoms, or duration. This is despite differences in age, cardiovascular risk factors, or peak troponin (Table 1). Accordingly, the chest pain characteristics in patients with MINOCA are indistinguishable from those with MICAD. However, compared to the MICAD patients, those with MINOCA were more likely to experience chest pain symptoms precipitated by emotional stress (5% vs. 11%, *p* < 0.001) and less likely to experience those precipitated by physical activity (39% vs. 31%, *p* < 0.05).

**Figure 3:**
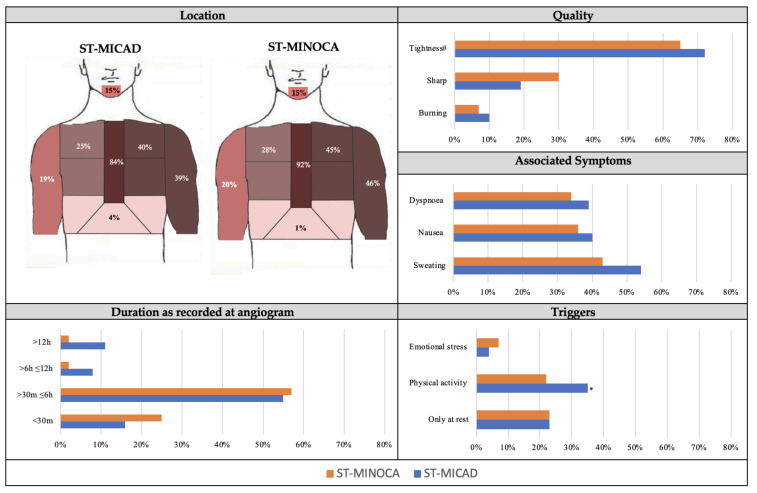
Chest pain characteristics of ST elevation MICAD (ST-MICAD) and ST elevation MINOCA (ST-MINOCA) patients. MICAD, myocardial infarction with coronary artery disease; MINOCA, myocardial infarction with non-obstructive coronary arteries; h, hours; m, minutes. # Tightness is a pooled analysis including the chest pain characteristics of tightness, heaviness and squeezing. * *p*-value <0.05.

Evaluating the subgroup with STEMI features, there were no significant differences in chest pain characteristics between patients with MICAD and MINOCA (Figure 3). However, chest pain precipitated by physical activity was more common in those with MICAD than MINOCA (35% vs. 22%, *p* < 0.05). Similarly, in the NSTEMI subgroup, there were no significant differences in chest pain characteristics between MICAD and MINOCA patients, except for the trigger of emotional stress, which was more prevalent in MINOCA patients compared to MI-CAD patients (12% vs. 5%, *p* < 0.05) (Supplementary Materials).

## 4. Discussion

Chest pain is a hallmark symptom of MI and is caused by inadequate blood supply to the myocardium. The underlying mechanisms of chest pain in MI are complex, and involve a combination of factors including inflammation, ischaemia, and nerve activation [1,10,11,12]. Utilising the unique CADOSA registry of consecutive patients who fulfilled the universal criteria for MI [1], the prevalence of MINOCA was consistent with other studies, featuring in 6% of all MI presentations [13]. For example, in a recent systematic review by the MINOCA global collaboration, the prevalence was 6% [14]. Additionally, similarly to previous studies [14,15], the MINOCA cohort were younger, more often women, and had fewer cardiovascular risk factors and lower peak troponin compared to MICAD patients. The MINOCA patients were also less likely to present with STEMI compared to MICAD patients. (Table 1). Fewer secondary cardioprotective therapies and cardiac rehabilitation program referrals were provided to MINOCA patients compared to MICAD, similar to previous studies [16,17]. This may be explained by the lack of a consensus strategy for treatment of MINOCA due to the lack of prospectively designed randomised therapeutic clinical trials; hence, guidelines are based on observational studies and expert opinion [13]. Nevertheless, it is concerning that these MINOCA patients are undertreated, as there have been studies showing the impact on their health status [18]. In a study by Grozinsky et al. [19], it was revealed that 25% of MINOCA patients have ongoing chest pain and a reduced quality of life at 12-month follow-up. This burden was at least as high as those with MICAD [19].

There were no differences in chest pain characteristics between those with MICAD or MINOCA. The MICAD and MINOCA cohorts were indistinguishable in relation to chest pain (i) location (central, 87% vs. 84%), (ii) quality (tightness, 72% vs. 73%), (iii) associated symptoms (dyspnea, 41% vs. 44%), and (iv) typical duration (>30 min ≤6 h, 49% vs. 50%) (Figure 2). Furthermore, when the cohort was restricted to those with STEMI (i.e., definitive ischaemic ECG changes with serial troponin changes), there remained no differences between the MICAD and MINOCA patients (Figure 3). Additionally, there were no differences in the NSTEMI patients (Supplementary Table S2). Thus, if MICAD patients are considered the benchmark for ischaemic chest pain, and those with MINOCA have indistinguishable chest pain features, then it could be concluded that they also have ischaemic chest pain. However, the cause of the myocardial ischaemia can differ considering the absence of obstructive CAD, and thus MINOCA patients require further diagnostic testing to determine the underlying pathogenesis causing their chest pain symptoms. This should include CMR imaging, as recommended by society position papers from the European Society of Cardiology [4], the Task Force for the Universal Definition of MI [1], and the American Heart Association [3]. CMR imaging is considered to be the gold standard in identifying the cause in approximately 90% of suspected MINOCA patients; it can confirm the presence of MI and exclude non-ischaemic causes. Its utility lies in (a) identifying the presence of MI on late gadolinium enhancement imaging; (b) evaluating left ventricular size and function; and (c) identifying (if performed within 2 weeks of presentation) the presence of myocarditis or takotsubo syndrome.

This study provides further support for the idea that MINOCA is a unique clinical ischaemic entity, with novel prospectively collected clinical data showing chest pain characteristics indistinguishable from those in patients with MICAD. Thus, concerns that patients with MINOCA are an arbitrary collection of patients with atypical chest pain with false-positive troponin are repudiated.

### Study Limitations

This study is unique, considering that it is a representative sample with consecutive patient recruitment that utilises data from direct patient interviews, and it has an appropriately defined MINOCA subgroup to compare to MICAD patients. However, despite these strengths, the study has important limitations that should be considered when interpreting the findings. Firstly, out-of-hospital cardiac arrest patients were excluded from the study, despite potentially being ischaemic in nature. These patients were excluded because the troponin changes could be the result of resuscitation efforts rather than ischaemia/infarction.

Secondly, exclusion of non-ischaemic causes for the suspected MI presentation was based upon the treating clinician’s diagnosis upon discharge. CMR imaging was not undertaken in all MINOCA patients; thus, the MINOCA cohort may potentially have included patients with undiagnosed myocarditis. However, routine cardiac magnetic resonance imaging in all MINOCA patients remains a challenge at most institutions due to availability, so the study does reflect routine practice. Moreover, it is noteworthy that the MINOCA patients were similar to those with MICAD with respect to chest tightness and associated symptoms.

## 5. Conclusions

This original prospective investigation of consecutive myocardial infarct patients provides compelling evidence that individuals with MINOCA exhibit chest pain characteristics that are clinically indistinguishable from those in patients with MICAD, with the only distinguishing factor being the absence of obstructive coronary artery disease on angiography. These findings strongly support the concept that MINOCA patients have ischaemic chest pain, thereby fulfilling the universal criteria for an acute MI in the context of myocardial injury. Certainly, they should not be disregarded as “false-positive infarcts” solely due to the absence of obstructive CAD.

Despite these significant findings, this study also highlights the need for further research to explore the underlying mechanisms and potentially related clinical features of MINOCA. Future studies should aim to investigate these aspects by utilising appropriate diagnostic tools and testing modalities. By doing so, a deeper understanding of the different subtypes and etiologies of MINOCA can be achieved, thereby contributing to the development of more tailored and effective therapeutic strategies for these patients.

## Figures and Tables

**Table 1:** Clinical characteristics of MICAD and MINOCA patients.

	MICAD		MINOCA		OR (95% of CI)	*p*
Baseline characteristics		(*n*)		(*n*)		
Age *	64 (20)	(5948)	59 (24)	(411)	-	<0.001
Female	26%	(1542)	56%	(230)	4.06 (3.30, 5.00)	<0.001
Indigenous	6%	(346)	5%	(22)	0.49 (0.31, 0.78)	0.003
Current smoking	37%	(2019)	28%	(110)	0.45 (0.35, 0.58)	<0.001
Hypertension	65%	(3790)	59%	(236)	0.79 (0.64, 0.99)	0.044
Hyperlipidaemia	62%	(3534)	54%	(215)	0.76 (0.61, 0.93)	0.009
Family Hx of CAD	42%	(2144)	40%	(150)	0.79 (0.63, 0.99)	0.039
Diabetes	33%	(1931)	19%	(78)	0.46 (0.35, 0.59)	<0.001
No chest pain in 2 weeks	48%	(2503)	57%	(216)	1.38 (1.11, 1.71)	<0.001
Prior MI	23%	(1293)	12%	(49)	0.56 (0.41, 0.77)	<0.001
Prior heart failure	6%	(359)	4%	(15)	0.59 (0.35, 1.01)	0.053
Prior valvular HD	2%	(105)	0.5%	(2)	0.40 (0.13, 1.28)	0.122
Prior PCI #	14%	(817)	5%	(19)	0.36 (0.24, 0.54)	<0.001
Prior CABG	8%	(444)	0%	(0)	-	
Current dialysis	2%	(124)	1%	(5)	0.39 (0.16, 0.98)	0.046
Prior stroke	8%	(468)	9%	(34)	1.16 (0.79, 1.69)	0.443
History of PAD	7%	(409)	5%	(18)	0.69 (0.42, 1.13)	0.136
Depression	13%	(652)	18%	(62)	1.14 (0.85, 1.54)	0.319
Peak troponin *	489 (1454)	(5259)	148 (339)	(346)	-	0.037
STEMI	43%	(2532)	19%	(78)	0.29 (0.23, 0.38)	<0.001
Discharge medications						
Aspirin	90%	(5322)	71%	(289)	0.25 (0.19, 0.32)	<0.001
Statin	89%	(5144)	66%	(267)	0.23 (0.18, 0.29)	<0.001
ACEI/ARB	82%	(4732)	60%	(245)	0.33 (0.26, 0.41)	<0.001
Beta blocker	66%	(3804)	36%	(145)	0.28 (0.23, 0.36)	<0.001
CCB	21%	(1224)	28%	(116)	1.49 (1.19, 1.88)	<0.001
Cardiac rehab	58%	(3119)	17%	(66)	0.15 (0.11, 0.20)	<0.001

Values are presented as percentages with numbers (%) or median with interquartile ranges. ACEI, angiotensin-converting enzyme inhibitor; ARB, angiotensin II receptor blocker; CABG, coronary artery bypass surgery; CAD, coronary artery disease; CCB, calcium channel blocker; Family Hx, Family history; MI, myocardial infarction; MICAD, myocardial infarction with coronary artery disease; MINOCA, myocardial infarction with non-obstructive coronary arteries; NSTEMI, non-ST elevation myocardial infarction; PAD, peripheral artery disease; PCI, percutaneous coronary intervention; STEMI, ST-elevation myocardial infarction. # Patients were considered MINOCA if the prior PCI was >6 months and no residual obstructive CAD on index admission angiography. * Age and peak troponin are displayed as median (inter quartile range (IQR)).

## Data Availability

The data presented in this study are available on request from the corresponding author. The data are not publicly available due to privacy and ethical restrictions.

## Supplementary Materials

The following supporting information can be downloaded at: https://www.mdpi.com/article/10.3390/jcm12144595/s1. Table S1: Definitions used for cardiovascular risk factors. Figure S1: Front page of CADOSA Data form. Figure S2: Chest pain characteristics of NST-MICAD and NST-MINOCA patients.

## References

[1] Thygesen K., Alpert J.S., Jaffe A.S., Chaitman B.R., Bax J.J., Morrow D.A., White H.D., ESC Scientific Document Group (2019). Fourth universal definition of myocardial infarction (2018). Eur. Heart J..

[2] Pasupathy S., Air T., Dreyer R.P., Tavella R., Beltrame J.F. (2015). Systematic review of patients presenting with suspected myocardial infarction and nonobstructive coronary arteries. Circulation.

[3] Tamis-Holland J.E., Jneid H., Reynolds H.R., Agewall S., Brilakis E.S., Brown T.M., Lerman A., Cushman M., Kumbhani D.J., Arslanian-Engoren C. (2019). Contemporary Diagnosis and Management of Patients with Myocardial Infarction in the Absence of Obstructive Coronary Artery Disease: A Scientific Statement from the American Heart Association. Circulation.

[4] Agewall S., Beltrame J.F., Reynolds H.R., Niessner A., Rosano G., Caforio A.L., De Caterina R., Zimarino M., Roffi M., Kjeldsen K. (2017). ESC working group position paper on myocardial infarction with non-obstructive coronary arteries. Eur. Heart J..

[5] Pasupathy S., Beltrame J.F. (2021). Refining the Role of CMR Imaging in MINOCA. JACC Cardiovasc. Imaging.

[6] Beltrame J.F. (2013). Assessing patients with myocardial infarction and nonobstructed coronary arteries (MINOCA). J. Intern. Med..

[7] Brindis R.G., Fitzgerald S., Anderson H.V., Shaw R.E., Weintraub W.S., Williams J.F. (2001). The American College of Cardiology-National Cardiovascular Data Registry (ACC-NCDR): Building a national clinical data repository. J. Am. Coll. Cardiol..

[8] Rao S.V., McCoy L.A., Spertus J.A., Krone R.J., Singh M., Fitzgerald S., Peterson E.D. (2013). An updated bleeding model to predict the risk of post-procedure bleeding among patients undergoing percutaneous coronary intervention: A report using an expanded bleeding definition from the National Cardiovascular Data Registry CathPCI Registry. JACC Cardiovasc. Interv..

[9] Pasupathy S., Tavella R., Beltrame J.F. (2017). Myocardial Infarction with Nonobstructive Coronary Arteries (MINOCA): The Past, Present, and Future Management. Circulation.

[10] Crea F., Liuzzo G. (2013). Pathogenesis of acute coronary syndromes. J. Am. Coll. Cardiol..

[11] Libby P., Theroux P. (2005). Pathophysiology of coronary artery disease. Circulation.

[12] De Araújo Gonçalves P., Ferreira J., Aguiar C., Seabra-Gomes R. (2005). TIMI, PURSUIT, and GRACE risk scores: Sustained prognostic value and interaction with revascularization in NSTE-ACS. Eur. Heart J..

[13] Lindahl B., Baron T., Erlinge D., Hadziosmanovic N., Nordenskjöld A., Gard A., Jernberg T. (2017). Medical Therapy for Secondary Prevention and Long-Term Outcome in Patients with Myocardial Infarction with Nonobstructive Coronary Artery Disease. Circulation.

[14] Pasupathy S., Lindahl B., Litwin P., Tavella R., Williams M.J.A., Air T., Zeitz C., Smilowitz N.R., Reynolds H.R., Eggers K.M. (2021). Survival in Patients with Suspected Myocardial Infarction with Nonobstructive Coronary Arteries: A Comprehensive Systematic Review and Meta-Analysis From the MINOCA Global Collaboration. Circ. Cardiovasc. Qual. Outcomes.

[15] Safdar B., Spatz E.S., Dreyer R.P., Beltrame J.F., Lichtman J.H., Spertus J.A., Reynolds H.R., Geda M., Bueno H., Dziura J.D. (2018). Presentation, Clinical Profile, and Prognosis of Young Patients with Myocardial Infarction with Nonobstructive Coronary Arteries (MINOCA): Results from the VIRGO Study. J. Am. Heart Assoc..

[16] Lawless M., Appelman Y., Beltrame J.F., Navarese E.P., Ratcovich H., Wilkinson C., Kunadian V. (2023). Sex differences in treatment and outcomes among myocardial infarction patients presenting with and without obstructive coronary arteries: A prospective multicentre study. Eur. Heart J. Open.

[17] Paolisso P., Bergamaschi L., Saturi G., D’Angelo E.C., Magnani I., Toniolo S., Stefanizzi A., Rinaldi A., Bartoli L., Angeli F. (2019). Secondary Prevention Medical Therapy and Outcomes in Patients with Myocardial Infarction with Non-Obstructive Coronary Artery Disease. Front. Pharmacol..

[18] Berg E., Agewall S., Brolin E.B., Caidahl K., Cederlund K., Collste O., Daniel M., Ekenback C., Jensen J., Y-Hassan S. (2022). Health-Related Quality-of-Life up to one year after myocardial infarction with non-obstructive coronary arteries. Eur. Heart J. Qual. Care Clin. Outcomes.

[19] Grodzinsky A., Arnold S.V., Gosch K., Spertus J.A., Foody J.M., Beltrame J., Maddox T.M., Parashar S., Kosiborod M. (2015). Angina Frequency after Acute Myocardial Infarction in Patients without Obstructive Coronary Artery Disease. Eur. Heart J. Qual. Care Clin. Outcomes.

