# Supplementary material for: Do Chest Pain Characteristics in Patients with Acute Myocardial Infarction Differ between Those with and without Obstructive Coronary Artery Disease?
Source: J Clin Med. 2023 Jul 10;12(14):4595. doi: 10.3390/jcm12144595 (PMC10380967; doi:10.3390/jcm12144595)
Supplement: Supplementary file 1 [file jcm-12-04595-s001.zip › jcm-2359017-supplementary.pdf]

Supplementary Table S1: Definitions used for cardiovascular risk factors

Risk factor	Definition
Hypertension	Prior medical diagnosis, or current use of antihypertensive agents.
Diabetes	Known history of diabetes with or without active utilization of diabetes medications
Hyperlipidaemia	Total cholesterol greater than 5.17 mmol/L; or LDL greater than or equal to 3.36 mmol/L; or, High-density lipoprotein (HDL) less than 1.03 mmol/L and for patients with known CAD, treatment is initiated if LDL is greater than 2.59 mmol/L
Smoking history	Current smoker: if the patients has been smoking cigarettes currently daily or non-daily.; Former smoker: the patient has not smoked cigarettes during the last year; No smoking history: if the patient has never smoked cigarettes
Family history	Direct relatives who have had any of the following at age less than 55 years for male relatives or less than 65 years for female relatives: Angina, acute MI, sudden cardiac death without obvious cause, coronary bypass grafting surgery (CABG) and PCI.

Supplementary Figure S1: Front page of CADOSA Data form.

CADOSA

Coronary Angiogram Database of South Australia
Diagnostic Catheterisation and Percutaneous Coronary Intervention Registry

FACILITY[1010]

○ CW	○ FMC	○ LMH	○ RAH	○ TQEH

Patient in Follow-up Study? [1020] ○ No ○ Yes

PART A: DEMOGRAPHICS

Surname [2000]:

First Name [2010]:

Middle Name [2020]:

Medicare No [2030]: |__||__||__||__||__||__||__||__||__||__||__| Patient UR [2040]: |__||__||__||__||__||__||__||__||__||__|

Post Code [3005]: |__||__||__||__| Date of Birth [2050]: |__||__|dd |__||__|mm |__||__||__||__|yyyy   Gender [2060]: ○ M ○ F

Postcode N/A [3006]: ☐

Ethnicity: ☐ Caucasian [2070] ☐ Indigenous/Torres Strait Islander [2071] ☐ Asian [2072] ☐ Hispanic [2073]

☐ African [2074] ☐ Sub-Continent [2075] ☐ Other [2076,2077] (specify) ___________________

PART B: EPISODE OF CARE and CHEST PAIN EVALUATION

Arrival to Cath Facility Date [3000]: |__||__|dd |__||__|mm 20|__||__|yyyy Time [3001]: |__||__|hh : |__||__|mm 24 hr

Referral Source [3010]: ○ Emergency Department ○ Admissions Office ○ Current In-patient ○ Transfer in From Other Acute Care Facility

Payer for Episode of Care [3020]: ○ Private Health Insurance ○ Medicare Only ○ Veteran Gold Card ○ Other

Transport to Cath Facility [3040]: ○ Self ○ SAAS ○ Air Ambulance ○ MedSTAR ○ Inter-hospital transfer – Air ○ Inter-hospital transfer – road

THE FOLLOWING QUESTIONS RELATE TO THE CHEST PAIN SYMPTOMS PROMPTING THIS DIAGNOSTIC CATHERTERISATION

Chest Pain Prompting this Diagnostic Catheterisation [3041]: ○ No ○ Yes ○ Unknown → If yes, complete below

Location of Chest Pain: (check all that apply)

☐ A[3042] ☐ B[3043] ☐ C[3044]
☐ D[3045] ☐ E[3046] ☐ F[3047]
☐ G[3048] ☐ H[3049] ☐ I[3050]
☐ J[3051] ☐ K[3052] ☐ L[3053]
☐ M[3054] ☐ N[3055] ☐ O[3056]
☐ P[3057] ☐ Q[3058] ☐ Other[3059]
☐ Unknown[3060]

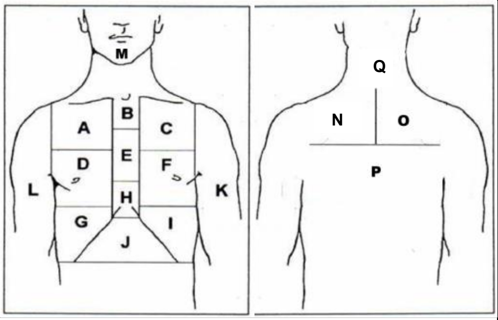

Quality of Chest Pain: ☐ Burning [3061] ☐ Squeezing [3062] ☐ Tightness [3063] ☐ Sharp [3064] ☐ Heavy [3065] ☐ Other [3066, 3067] ____________ ☐ Unknown [3068]

Precipitating Factors: ☐ Exertion [3069] ☐ Meals [3070] ☐ Emotional Stress [3071] ☐ Cold Weather [3072] ☐ Nocturnal [3073] ☐ Lying Down [3074] ☐ Pleuritic [3075] ☐ Only at Rest [3076] ☐ Other [3077, 3078] _________________ ☐ Unknown [3079]

Relieving Factors: ☐ Rest [3080] ☐ Nitrates (<5 mins) [3081] ☐ Nitrates (> 5 mins) [3082] ☐ Antacids [3083] ☐ Other [3084, 3085] ________________________ ☐ Unknown [3086]

Associated Symptoms: ☐ Tachypnea [3087] ☐ Rapid Palpitations [3088] ☐ Pre-syncope/syncope [3089] ☐ Post-pain fatigue [3090] ☐ Nausea/vomiting [3091] ☐ Sweating [3092] ☐ Chest Wall Tenderness [3093] ☐ Other [3094, 3095] ________________________ ☐ None [3096] ☐ Dyspnea [3097] ☐ Unknown [3098]

Typical Duration [3099]: ○ ≤ 15 seconds ○ > 15 seconds ≤ 15 minutes ○ > 15 minutes ≤ 30 minutes ○ > 30 minutes ≤ 60 minutes ○ > 60 minutes ≤ 2 hours ○ > 2 hours ≤ 6 hours ○ > 6 hours ≤ 12 hours ○ > 12 hours ○ Unknown

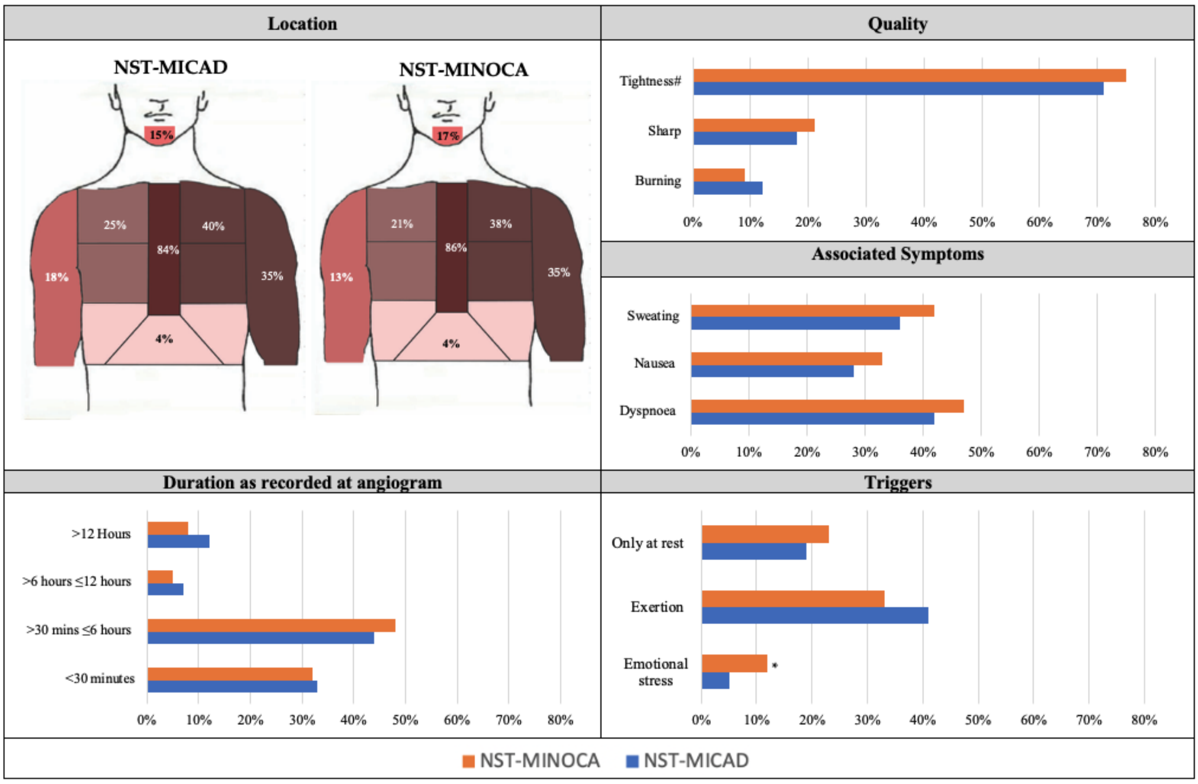

Supplementary Figure S2: Chest pain characteristics of NST-MICAD and NST-MINOCA patients. NST - MICAD, Non ST elevation myocardial infarction with coronary artery disease; NST-MINOCA, Non ST elevation myocardial infarction with non-obstructive coronary arteries; h, hours; m, minutes. #Tightness is a pooled analysis including chest pain characteristics: tightness, heaviness and squeezing. * p-value <0.05.
